# Effects of Enhanced Recovery After Surgery (ERAS) on Perioperative Outcomes in Shoulder Arthroplasty

**DOI:** 10.7759/cureus.92284

**Published:** 2025-09-14

**Authors:** Alexander M Wong, Hayden L Hofmann, Gage A Guerra, Jonathan L Le, Zachary Singh, Frank Petrigliano, Abdullah Foad

**Affiliations:** 1 Orthopedic Surgery, University of Southern California Keck School of Medicine, Los Angeles, USA; 2 Orthopedic Surgery, University of California Los Angeles, Los Angeles, USA; 3 Orthopedic Surgery, Morrison Community Hospital, Morrison, USA

**Keywords:** anatomic total shoulder arthroplasty, complication rates, enhanced recovery after surgery, in-hospital mortality, perioperative outcomes, reverse total shoulder arthroplasty

## Abstract

Background and objective

Enhanced Recovery After Surgery (ERAS) is a multidisciplinary, evidence-based management pathway to decrease length of stay (LOS) and complication rates. However, there is minimal research on ERAS regarding total shoulder arthroplasties (TSA). This study aimed to analyze the effects of the ERAS protocol on perioperative outcomes in patients who underwent elective, inpatient TSAs.

Methods

A retrospective analysis was conducted using the Premier Health Database. Patients who underwent an elective anatomic TSA (aTSA) or reverse TSA (rTSA) were categorized into ‘Low ERAS’, ‘Medium ERAS’, or ‘High ERAS’ based on the number of billable ERAS criteria. Mixed model logistic regressions yielded odds ratios (ORs) and 95% confidence intervals (CIs). An area under the receiver operating characteristic curve (AUROC) and a Youden index were calculated to determine the optimal cutoff point for the best predictive model.

Results

A total of 74,158 patients were identified. Of them, 15,285 (20.6%) patients were ‘High ERAS’, 21,809 (29.4%) were ‘Medium ERAS’, and 37,064 (50.0%) were ‘Low ERAS.’ ‘High ERAS’ patients had significantly decreased ORs of experiencing any complication (OR=0.78, p<0.001), cardiopulmonary complications (OR=0.75, p<0.001), in-hospital mortality (OR=0.054, p=0.0040), and blood transfusions (OR=0.58, p<0.0001), and a shorter LOS from 2.19 days to 1.84 days (p<0.0001). The AUROC for the number of ERAS criteria received and in-hospital mortality was 0.75 with an optimal cutoff point of four ERAS criteria.

Conclusions

TSAs are not an exception to the promising benefits of the ERAS protocol. Efforts should be made to create an official ERAS protocol in TSAs and help hospitals allocate resources to make its implementation possible.

## Introduction

Enhanced Recovery After Surgery (ERAS) is a multidisciplinary, evidence-based management pathway that has been widely adopted by medical subspecialties, including hepatobiliary surgery, gastroenterology, anesthesiology, gynecology, oncology, and urology [1]. The primary objective of ERAS is to maintain normal physiology perioperatively to minimize the stress of surgery [2]. Current ERAS protocols are stratified into preoperative, intraoperative, and postoperative phases, and generally include preoperative counseling, antimicrobial prophylaxis, maintenance of euvolemia, postoperative nausea/vomiting (PONV) prophylaxis, multimodal opioid-sparing analgesia, early mobilization, and early return to normal diet [2]. This multifaceted management of the patient requires collaboration between several providers of the patient’s care team [3,4]. Coordination and commitment of the staff are crucial to implement a standardized, yet patient-centered, protocol that will quicken recovery and improve the patient experience. Across multiple surgical specialties, implementation of ERAS criteria was found to significantly decrease length of stay (LOS), complication rates, cost, and mortality, and improve patient satisfaction [5-7].

ERAS protocol in orthopedic surgery has demonstrated great promise, as it has been shown to significantly reduce both LOS and postoperative complication rates in lumbar spinal fusion, hip fracture repair, total hip arthroplasty (THA), and total knee arthroplasty (TKA) [5,8,9]. Recently, the ERAS Society outlined protocols specific to THA and TKA [10]. These guidelines focus on standardizing an anesthetic protocol utilizing general anesthesia or neuraxial techniques, using local infiltration analgesia over nerve blocks, minimizing blood loss with tranexamic acid (TXA), and abstaining from the use of a Foley catheter and wound drain [10]. One study found that the use of regional anesthesia, multimodal analgesia, TXA, Day zero antiemetics, Day zero steroids, Day zero or one physical therapy, and absence of a Foley catheter and wound drain catheter significantly lowered complications, mortality, and LOS in THA and TKA patients [5].

Despite the widespread study and implementation of the ERAS protocol in orthopedic procedures, there is scarce literature exploring outcomes after the ERAS protocol in total shoulder arthroplasties (TSA). However, given the benefits of the ERAS protocol in THA/TKA, further research in TSA is warranted, as both incidence rates of TSAs performed and patient comorbidities are on the rise. One study showed a 94.6% increase in TSA incidence rates between 2011 and 2017 compared to 23.3% and 12.5% for THA and TKA, respectively [11]. TSA incidence rates are also projected to continue outpacing TKA and THA, with one model estimating a 235% increase in annual volume through 2025 [11]. This surge in TSA rates is largely due to the advent of the reverse total shoulder arthroplasty (rTSA) in 2004, which has exceeded anatomic shoulder arthroplasty (aTSA) since 2014 and is expected to continue increasing at a much greater rate [11,12].

TSAs are also most commonly performed on the elderly population, who have a higher prevalence of comorbidities, and are projected to continue dominating demand [13,14]. Studies have shown that comorbidities, such as obesity, diabetes, smoking, and COPD, are increasing in TSAs and cause increased complication rates, reoperation rates, and LOS [15-17]. Indeed, revision surgeries following TSAs are on the rise, which have increased 392% from 2002 to 2017. This steep rise in revision surgery rates translates to a 685% increase in national cost [18]. Accordingly, there must be advancements in protocols for TSAs. The current study aims to analyze the potential effects of the ERAS protocol on outcomes in patients who underwent an elective inpatient TSA. We hypothesize that an increased utilization of ERAS criteria will be associated with reduced complication rates, blood transfusion requirements, in-hospital mortality, and LOS following an elective, inpatient aTSA or rTSA.

This article was previously presented as a meeting abstract at the 2024 Orthopedic Research Society Meeting on February 5, 2024, in Long Beach, California.

## Materials and methods

Data were obtained from the Premier Healthcare Database (PHD) by Premier Applied Sciences, Premier Inc. This database includes over 127 million inpatient visits and represents approximately 25% of all inpatient admissions in the United States; 1,113 different hospitals contribute to the Premier Healthcare Database, predominantly from the South region (42%) and with a relatively even distribution from the Northeast, Midwest, and West regions (15%, 26%, and 18%, respectively). The South includes states such as Alabama, Arkansas, and Georgia. The Northeast includes states such as New York, New Jersey, and Maine. The Midwest includes states such as Iowa, Illinois, Wisconsin, and Michigan. The West includes states such as California, Oregon, and Washington. Hospital-level encounter data is claims-based and obtained from the uniform billing form (UB-04). Hospital-level encounter data includes characteristics such as attending physician specialty, point of origin, type of admission, and discharge status. Service and patient-level data are provided through the International Classification of Diseases, Ninth Revision (ICD-9) Diagnosis codes and through the PHD Chargemaster, which is a comprehensive list of all billable items to the hospital, patient, or insurance [19].

Patients who underwent an aTSA or rTSA were identified using ICD-9 codes 81.80 (total shoulder arthroplasty) and 81.88 (reverse total shoulder arthroplasty). Exclusion criteria were as follows: patients with missing demographic data or discharge data, cases described as outpatient procedures, admission types classified as non-elective, and cases with ICD-9 codes for both aTSA or rTSA.

ERAS criteria were identified following the protocol outlined by the ERAS Society for THA and TKA. However, only criteria within the PHD billing sheet could be included. Nine ERAS criteria were then selected: (1) regional anesthesia; (2) multimodal analgesia; (3) Day zero postoperative nausea/vomiting prophylaxis; (4) Day zero or one venous thromboembolism (VTE) prophylaxis; (5) Day zero or one physical therapy; (6) TXA; (7) absence of a Foley catheter; (8) absence of a wound drain; and (9) direct discharge to home. Regional anesthesia included a suprascapular nerve block, axillary nerve block, or brachial plexus nerve block, either as a single injection or a continuous infusion catheter. Multimodal analgesia included oral, parenteral, or suppository formulations of acetaminophen, NSAIDs, gabapentin, pregabalin, and COX-2 inhibitors. PONV prophylaxis included an oral or parenteral formulation of aprepitant, dolasetron, granisetron, ondansetron, palonosetron, perphenazine, metoclopramide, and corticosteroids (cortisone, fludrocortisone, prednisone, and triamcinolone). VTE prophylaxis included oral, parenteral, or suppository formulations of aspirin, rivaroxaban, enoxaparin, coumadin, and apixaban. TXA included oral or parenteral formulations. Medications were identified through the PHD ChargeMaster.

The frequency of each number of ERAS criteria received was obtained, and cutoff values were defined using tertiles, where Q1=5 and Q3=7. Patients were subsequently categorized based on the number of ERAS criteria received throughout their hospitalization. ‘Low ERAS’, ‘Medium ERAS’, and ‘High ERAS’ corresponded to patients who received <6, 6, or >6 ERAS criteria, respectively. The primary outcomes were: any complication, cardiopulmonary complications, in-hospital mortality, blood transfusion requirements, and LOS. ‘Any complication’, ‘cardiopulmonary complications’, and ‘blood transfusions’ were defined and obtained through ICD-9 codes. Only complications with an ICD-9 code “not present on admission” were included for ‘any complication’ and ‘cardiopulmonary complications.’ ‘In-hospital mortality’ was obtained from discharge status described as “expired”, “expired hospice medical facility”, and “expired hospice unknown.” Patients who expired in hospice were included since the decision to transition to hospice care reflects the poor prognosis of the patient and more accurately includes patients who became critical postoperatively. Additional variables studied include age, sex, race, insurance type, surgery type, and surgery year. Race was stratified into White, Black, or Other. Insurance type was divided into commercial, Medicare, Medicaid, other government payers, uninsured, other, or unknown. 

Descriptive statistics for demographic variables were obtained for each of the three groups (‘Low ERAS’, ‘Medium ERAS’, and ‘High ERAS’). Continuous variables and binary variables were compared to ERAS categories throughout hospitalization using ANOVA (age, surgery year) and chi-squared tests (gender, race, insurance, surgery type), respectively. A logistic regression was conducted to compare the use of each of the nine individual ERAS criteria variables between ‘Low ERAS’, ‘Medium ERAS’, and ‘High ERAS’ categories. A mixed model logistic regression measured the association between ‘Low’/ ‘Medium’/ ‘High ERAS’ groups and outcomes using a random intercept to account for variations between hospitals. The same technique was employed to determine the effect of all nine individual ERAS criteria on outcomes. A linear regression was performed to determine the relationship between ERAS categories and LOS. Odds ratios (OR) and 95% confidence intervals (CI) were obtained for binary outcomes. An area under the receiver operating characteristic curve (AUROC) was calculated for each complication, and a Youden index was calculated to determine the optimal cutoff point for the best predictive model. All statistical analyses were performed in R and Stata. Significance was set at p<0.05.

## Results

On examining data collected from 1,113 hospitals, we identified 74,158 records of patients who underwent aTSAs or rTSAs from 2010 to 2015. This cohort consisted of 42,016 aTSA (56.7%) and 32,142 rTSA (43.3%) patients. Nine ERAS components were identified, with patients receiving a median number of six components. Notably, 15,285 (20.6%) of patients were subject to at least seven ERAS components (‘High ERAS’), while 21,809 (29.4%) and 37,064 (50.0%) were administered six components and fewer than six components (‘Medium ERAS’ and ‘Low ERAS’, respectively). Detailed demographic variable breakdowns based on ERAS use levels are presented in Table 1. The most frequently applied ERAS components included the avoidance of a wound drain, the avoidance of a Foley catheter, and Day zero PONV prophylaxis. Conversely, the least applied ERAS components were regional anesthesia and TXA. Older patients and white patients were significantly more likely to receive high levels of ERAS components, as well as those with commercial payor insurance (p<0.0001).

**Table 1:** Demographic and study variables by High, Medium, and Low ERAS patient groups Data are presented as n (%) unless otherwise indicated. χ² and F-values are presented for chi-squared and ANOVA statistical tests, respectively ERAS: Enhanced Recovery After Surgery; ANOVA: analysis of variance; TSA: total shoulder arthroplasty; rTSA: reverse total shoulder arthroplasty

Study variables	High ERAS (n=15 285)	Medium ERAS (n=21 809)	Low ERAS (n=37 064)	P-value	Test statistic (χ²/F)
ERAS variables					
Regional anesthesia	5169 (33.82)	3829 (17.56)	3121 (8.42)	<0.0001	χ²=1293.25
Multimodal analgesia	14,406 (94.25)	17,659 (80.56)	18,009 (48.59)	<0.0001	χ²=1416.01
Tranexamic acid	4021 (26.31)	1901 (8.72)	1264 (3.41)	<0.0001	χ²=2072.60
Antiemetics, Day 0 (N/V)	14,889 (97.41)	19,547 (89.63)	22,921 (61.84)	<0.0001	χ²= 817.93
Venous thromboembolism prophylaxis, Day 0 or Day 1	13,739 (89.89)	14,856 (68.12)	14,543 (39.24)	<0.0001	χ²=2410.67
Physical therapy, Day 0 or Day 1	14,133 (92.46)	17,175 (78.75)	20,625 (55.65)	<0.0001	χ²=1283.30
Foley absence	14,818 (96.94)	19,624 (89.98)	27,934 (75.37)	<0.0001	χ²=656.45
Wound drain absence	15,150 (99.12)	21,282 (97.58)	34,267 (92.45)	<0.0001	χ²=120.52
Home discharge	13,480 (88.19)	15,071 (69.10)	19,702 (53.16)		χ²=1846.83
Age, continuous	68.54	69.25	70.01	<0.0001	F=7.14
Sex				<0.0001	χ²=40.85
Female	8182 (53.53)	12,405 (56.88)	21,816 (58.86)		
Male	7103 (46.47)	9404 (43.12)	15,248 (41.14)		
Race				<0.0001	χ²=25.35
White	13,499 (88.31)	18,905 (86.69)	31,388 (84.68)		
Black	598 (3.91)	893 (4.09)	1733 (4.68)		
Other	1188 (7.78)	2011 (9.22)	3943 (10.64)		
Insurance				0.00050	χ²=25.13
Commercial	3714 (24.30)	4949 (22.69)	7408 (19.99)		
Medicare	10,343 (67.67)	15,203 (69.71)	26,963 (72.75)		
Medicaid	450 (2.94)	557 (2.55)	823 (2.22)		
Uninsured	2 (0.00)	2 (0.00)	3 (0.00)		
Other government payors	194 (1.27)	251 (1.15)	460 (1.24)		
Other	457 (2.99)	629 (2.88)	1021 (2.75)		
Unknown	125 (0.83)	218 (1.02)	386 (1.05)		
Procedure-related					
Year of surgery				<0.0001	F=131.02
2010	764 (5.16)	1742 (8.18)	4506 (12.37)		
2011	1363 (9.21)	2581 (12.13)	5707 (15.67)		
2012	1944 (13.14)	3389 (15.92)	6628 (18.20)		
2013	2949 (19.93)	4324 (20.32)	7046 (19.34)		
2014	4160 (28.11)	5199 (24.43)	7417 (20.36)		
2015	3618 (24.45)	4049 (19.02)	5119 (14.06)		
Type of surgery				0.47	χ²=0.0060
TSA	8717 (57.03)	12,429 (56.99)	20,870 (56.31)		
rTSA	6568 (42.97)	9380 (43.01)	16,194 (43.69)		
Academic center				0.052	χ²=3.78
Yes	6593 (43.1%)	9186 (42.1%)	15,096 (40.7%)		
No	8692 (56.9%)	12,623 (57.9%)	21,698 (59.3%)		
Location description				<0.0001	χ²=56.42
Urban	13,422 (87.8%)	19,686 (90.3%)	34,025 (91.8%)		
Rural	7025 (12.2%)	2123 (9.7%)	3039 (8.2%)		

**Figure 1:**
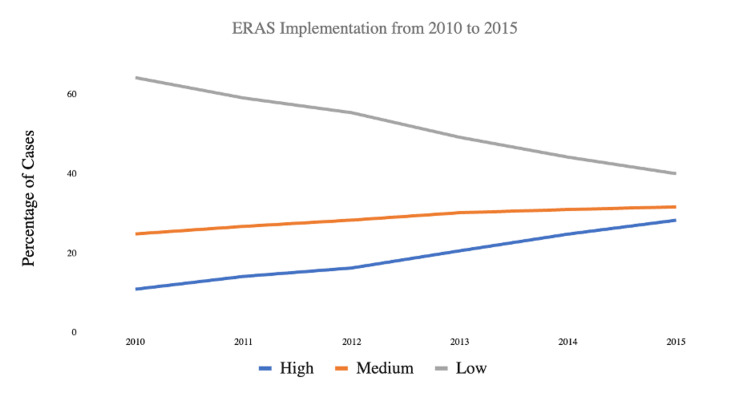
ERAS implementation trends from 2010 to 2015 by level ERAS: Enhanced Recovery After Surgery

**Figure 2:**
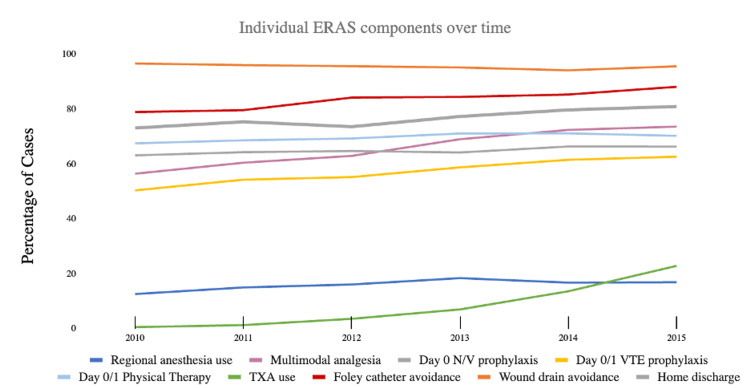
ERAS implementation trends from 2010 to 2015 by individual component ERAS: Enhanced Recovery After Surgery; TXA: tranexamic acid; VTE: venous thromboembolism

The relevant trends are highlighted in Figure 1. ‘High ERAS’ showed the strongest positive trends over these five years. Across all aTSA and rTSA procedures, 3,618 (28.3%) patients received ‘High ERAS’ components in 2015 compared to 764 (10.9%) patients in 2010. Conversely, ‘Low ERAS’ showed the strongest negative trends with 5,119 (40.0%) patients receiving ‘Low ERAS’ components in 2015 compared to 4,506 (64.3%) patients in 2010. Of individual ERAS components, the administration of TXA had the strongest positive trend with a 22.37% increase from 2010 to 2015 (Figure 2). Multimodal analgesia usage and Day zero/one VTE prophylaxis use demonstrated strong positive trends as well, with a 17.32% and 12.40% increase from 2010 to 2015, respectively. Wound drain avoidance decreased by 0.88% from 2010 to 2015.

**Figure 3:**
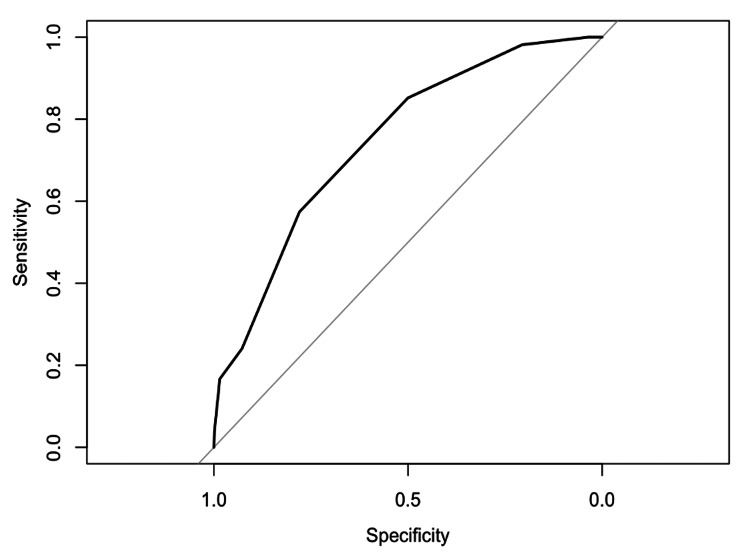
ROC of the number of ERAS criteria received vs. in-hospital mortality AUROC=0.75. Youden index=4.0. AUROC: area under the receiver operating characteristic curve; ERAS: Enhanced Recovery After Surgery

Table 2 highlights the absolute difference between perioperative patient outcomes and ‘High’, ‘Medium’, or ‘Low’ ERAS. The corresponding odds ratios are displayed in Table 3. ‘High ERAS’ patients had significantly decreased ORs of experiencing any complication (OR=0.78, p<0.0001), cardiopulmonary complications (OR=0.75, p<0.0001), in-hospital mortality (OR=0.054, p=0.0040), and blood transfusions (OR=0.58, p<0.0001). ‘Medium ERAS’ and ‘High ERAS’ patients also had a shorter LOS from 2.19 days to 2.01 and 1.84 days, respectively (p<0.0001). Avoidance of Foley catheters protected against all complications examined (p<0.0001). Day zero/one physical therapy was significantly associated with decreased complication rates. Lastly, TXA was significantly associated with decreased transfusion rates and decreased complication rates. However, multimodal analgesia and Day zero/one VTE prophylaxis were associated with an increase in ‘any complication’ (OR=1.24, p<0.001; OR=1.17, p<0.001, respectively) and ‘cardiopulmonary complications’ (OR=1.31, p<0.001; OR=1.19, p<0.001, respectively) (Table 4). Interventions promoting early mobility (i.e., home discharge, early physical therapy, and avoidance of a Foley catheter) and the use of multimodal analgesia have the strongest effect estimates regarding associations with outcomes. The AUROC for the number of ERAS criteria received and in-hospital mortality was 0.75 with an optimal-cutoff point of four ERAS criteria (Figure 3).

**Table 2:** Absolute differences between enhanced ERAS groups and perioperative outcomes ERAS: Enhanced Recovery After Surgery

Outcome	Unadjusted (absolute differences)					
	Low ERAS		Medium ERAS		High ERAS	
	N	%	N	%	N	%
Any complication	1919	53.39	1061	29.52	614	17.09
Cardiopulmonary complication	1312	54.24	683	28.23	424	17.53
In-hospital mortality	46	85.19	7	12.96	1	1.85
Blood transfusion need	594	59.64	277	27.81	125	12.55
Length of stay	2.19		2.01		1.84	

**Table 3:** Relative effects from mixed-effects regression evaluating the (adjusted) association between ‘High’ or ‘Medium’ (compared with ‘Low’) ERAS Relative effects are reported as OR for binary outcomes ERAS: Enhanced Recovery After Surgery; OR: odds ratios; CI: confidence interval

Outcome	Relative effects				
	Low ERAS	Medium ERAS		High ERAS	
		OR (95% CI)	P-value	OR (95% CI)	P-value
Any complication	Reference	0.93 [0.85-1.00]	0.077	0.78 [0.70-0.86]	<0.0001
Cardiopulmonary complication	Reference	0.83 [0.75-0.92]	0.00031	0.75 [0.66-0.85]	<0.0001
In-hospital mortality	Reference	0.26 [0.12-0.59]	0.0012	0.054 [0.0073-0.39]	0.0040
Blood transfusion need	Reference	0.70 [0.59-0.83]	<0.0001	0.58 [0.46-0.74]	<0.0001
Length of stay	Reference		<0.0001		<0.0001

**Table 4:** Results from mixed-effects regression evaluating the (adjusted) association between individual ERAS components and outcomes ^*^ORs not calculated due to zero events in one or more groups Relative effects are reported as OR for binary outcomes ERAS: Enhanced Recovery After Surgery; OR: odds ratios; CI: confidence interval; PONV: postoperative nausea/vomiting

ERAS variables	Any complication		Cardiopulmonary complication		Transfusion		In-hospital mortality	
	OR (95% CI)	P-value	OR (95% CI)	P-value	OR (95% CI)	P-value	OR (95% CI)	P-value
Regional anesthesia	0.98 [0.91-1.06]	0.64	1.04 [0.95-1.14]	0.44	1.02 [0.87-1.21]	0.79	0.40 [0.14-1.10]	0.076
Multimodal analgesia	1.24 [1.17-1.32]	<0.001	1.31 [1.21-1.41]	<0.001	1.52 [1.31-1.77]	<0.001	0.72 [0.41-1.28]	0.26
Tranexamic acid	0.88 [0.80-0.97]	0.01	0.95 [0.85-1.08]	0.44	0.33 [0.23-0.45]	<0.001	1.44 [0.61-3.41]	0.41
PONV prophylaxis, Day 0	0.95 [0.89-1.01]	0.12	0.92 [0.84-0.99]	0.04	0.94 [0.81-1.10]	0.42	1.08 [0.58-1.99]	0.81
Physical therapy, Day 0 or Day 1	0.91 [0.86-0.97]	0.003	0.88 [0.81-0.95]	0.001	0.89 [0.78-1.03]	0.89	0.28 [0.16-0.47]	<0.001
Foley absence	0.74 [0.68-0.79]	<0.001	0.80 [0.73-0.87]	<0.001	0.51 [0.44-0.58]	0.51	0.39 [0.22-0.69]	0.001
Wound drain absence	1.03 [0.91-1.17]	0.62	0.93 [0.80-1.08]	0.31	0.83 [0.64-1.06]	0.13	0.98 [0.35-2.77]	0.97
Home discharge	0.43 [0.41-0.45]	<0.001	0.45 [0.42-0.48]	<0.001	0.33 [0.29-0.38]	<0.001	—^*^	—^*^
VTE prophylaxis, Day 0/1	1.17 [1.11-1.24]	<0.001	1.19 [1.10-1.28]	<0.001	1.12 [1.04-1.35]	0.42	0.89 [0.52-1.55]	0.89

## Discussion

This study investigated the impact of ERAS implementation on perioperative complications and LOS in 74,158 patients who underwent inpatient, elective aTSAs and rTSAs. Overall, patients receiving ‘High ERAS’ components demonstrated better outcomes with significantly lower complication rates, mortality rates, LOS, and need for blood transfusions (Table 3). Figure 1 also demonstrates a strong upward trend of ‘High ERAS’ level utilization from 2010 to 2015, although levels of both ‘Low ERAS’ and ‘Medium ERAS’ utilization remained higher. The lower implementation of ERAS criteria may be due to the resource-intensive nature of the ERAS protocol, which requires more medications and interdisciplinary staff to oversee the patient pre-, peri-, and postoperatively. Within the ERAS criteria, Day zero/one physical therapy and the absence of a Foley catheter were particularly associated with lower odds of complications (Table 4).

Our findings are consistent with other studies that explored outcomes after TSA in novel fast-track rehabilitation programs similar to ERAS. Grosh and colleagues [3] found a decreased LOS after their Shoulder Arthroplasty enhanced Recovery Protocol (ShARP), which focused on pre-operative education, multimodal analgesia with an interscalene block, antifibrinolytics, and multimodal PONV prophylaxis. An alternative enhanced recovery pilot program by Morgan et al. found decreased LOS and complication rates after preoperative education, nutritional optimization, regional anesthesia, multimodal analgesia, and early physical therapy. The study’s 0.49-day decrease in LOS is similar to our decrease of 0.35 days [20]. Our study thus elaborates on previous enhanced recovery program studies in TSA by studying the effect of ERAS protocol on a more comprehensive list of complications in addition to LOS. However, our findings are more generalizable, with data coming from over 1,000 hospitals in all regions of the United States with various patient populations rather than a single hospital [19].

Our results are also consistent with studies that investigated ERAS-specific protocols in total joint arthroplasties. Memtsouidis et al. found a decreased rate of complications, in-hospital mortality, and transfusions in THA/TKA patients who received six or more ERAS criteria. They also found a decrease in mean LOS from three days to two days in patients who received six or more ERAS criteria [5]. Similarly, our study found decreased complication rates, in-hospital mortality, and blood transfusions in patients who received six or more ERAS criteria, as well as a decreased mean LOS from 2.19 to 1.84 days (Table 2). However, we introduced more ERAS criteria into our study to include Day zero/one VTE prophylaxis and direct discharge to home.

We found that Day zero/one physical therapy and Foley catheter avoidance were among the most implemented ERAS criteria that decreased the risk of complications. While there is currently no consensus on the timing of range of motion (ROM) exercises, immediate postoperative rehabilitation in both aTSAs and rTSAs has been shown to decrease pain scores and increase function with no significant effects on long-term outcomes [21,22]. Early mobilization is thus crucial for patients to quickly return to normal ADLs. Physical therapy is also important in mitigating further consequences associated with bed rest, such as insulin resistance, thromboembolic events, and decompensation of cardiopulmonary and musculoskeletal systems [23].

Postoperative complications may also arise from prolonged indwelling urinary catheters, which are a well-documented cause of urinary tract infections (UTI). In total joint arthroplasties (TJA), one study showed that patients returned to the hospital at a significantly higher rate if they intraoperatively received a Foley catheter [24]. While urinary retention may be common after shoulder surgery, Crain et al. concluded that Foley catheters did not significantly impact these rates [24]. Urinary catheters can thus be largely avoided to mitigate UTIs and their documented complications, including urosepsis, delirium, and cardiovascular events [25,26]. The beneficial effects of early physical therapy and Foley catheter avoidance explain our findings that these ERAS criteria independently lower the complications examined.

However, our study also found that multimodal analgesia and VTE prophylaxis carried significantly increased odds of experiencing perioperative complications (Table 4). This may be attributed to the inherent limitations of a retrospective study, as we could not assess when medications were given in the timeline of their hospitalization course. We speculate that some patients may have received these medications in response to a complication rather than as a preventive measure. Thus, the odds ratios for medications that are used to prevent as well as treat complications may be skewed.

The ERAS criteria that had the lowest administration were regional anesthesia and TXA. A long-lasting peripheral nerve block (PNB) has been shown to enhance the effects of multimodal analgesia and reduce postoperative pain and opioid use, making it superior to general anesthesia and a fundamental pillar in many clinical pathways for TJA [3,27,28]. While we found only modest use of regional anesthesia (Table 1), this may be due to hesitancy to administer a PNB due to the risk of permanent nerve injury, inconsistent results, or delay of safe mobilization [3,10]. TXA use may have been low during our study period, as the literature on TXA in TJAs is scarce before 2010. The steep increase in TXA during this time frame reflects the growing literature in TJAs, and specifically TSAs, towards 2015 (Figure 2).

Of the individual ERAS criteria, TXA and multimodal analgesia had the greatest increase in use from 2010 to 2015, rising 23.3% and 17.2%, respectively (Figure 2). Recently, TXA has been widely studied in aTSAs and rTSAs due to the high risk of perioperative blood loss, with blood transfusion rates ranging from 4.3% to 43% [27]. One meta-analysis found a decrease in blood transfusions from 2006 to 2018, attributing it to the growing acceptance of TXA and its effect on lowering blood transfusion rates [15,27]. Our surge in TXA use corroborates this finding. Multimodal analgesia is a central pillar of ERAS criteria to combat opioid use for postoperative pain management, which can lead to short- and long-term side effects such as nausea, constipation, sedation, and addiction. Non-opioid analgesics in aTSAs and rTSAs have been shown to significantly reduce postoperative opioid use while also lowering postoperative zero pain scores and LOS, prompting a recommendation for multimodal analgesia use by the American Society of Anesthesiologists in 2012 [29]. We observed the largest increase in multimodal analgesia use from 7,527 patients (62.4%) in 2012 to 9,881 patients (69.0%) in 2013 after this recommendation.

ERAS categories of ‘Low’, ‘Medium’, and ‘High’ had the greatest discriminatory ability for in-hospital mortality, quantified by the area under the receiver operating characteristic curve (AUROC) of 0.75 (Figure 3). The optimal cutoff was four ERAS criteria. Thus, receiving approximately half of the ERAS criteria decreases the risk of in-hospital mortality. This finding is supported by the low odds ratios of 0.26 and 0.053 for in-hospital mortality and ‘Medium ERAS’ and ‘High ERAS’, respectively (Table 3). This cut-off value can help physicians properly allocate resources and optimize care for patients undergoing an aTSA or rTSA who may be at higher risk for life-threatening complications.

Limitations

ERAS protocols selected for the study were only the billable items found in the PHD chargemaster. Other ERAS criteria to consider, such as preoperative nutrition or maintenance of fluid volume and normothermia, may have had an underlying influence on patient outcomes. The context under which ERAS interventions were given could not be discerned due to the nature of our retrospective database study. Furthermore, our categories of ‘High ERAS’, ‘Medium ERAS’, and ‘Low ERAS’ treat each ERAS criterion with equal weight and entail a wide variety of nerve blocks and combinations and dosages of medications that patients could have received. Certain techniques or medication combinations may positively or negatively interact and thus impact outcomes. While the positive impact of medications, such as multimodal analgesia and TXA, is widely established, there is no consensus on the optimal route, dosage, and timing of administration [4,20,29,30]. Thus, further prospective studies are needed to control for the heterogeneity of ERAS criteria implementation.

Future studies should also investigate associations between ERAS protocols and the rate of revision surgeries and long-term complications, as our study focused on perioperative complications following aTSA and rTSA. Future prospective studies that can further control for variables such as surgeon volume and preferences, institutional protocols, patient comorbidities, and patient socioeconomic status would be useful. Additionally, further analysis with more current data would be useful. The increased usage of regional anesthesia and TXA, which were modestly used in our study, is now highly utilized for their efficacy in reducing complications and improving patient recovery. Further analysis with current data would be helpful to see how this greater use affects patient recovery in the ERAS pathway. Finally, similar studies should be performed once an official TSA ERAS protocol is released.

In conclusion, we determined that an increase in the utilization of the ERAS protocol was associated with a decreased risk of complications, in-hospital mortality, and blood transfusions, and decreased the LOS in patients who underwent an inpatient, elective aTSA or rTSA. The ERAS protocol is promising in TSAs and warrants further studies, given the increasing demand for TSAs and the increasing age and number of comorbidities in patients who are undergoing these procedures.

## Conclusions

ERAS is a well-established, evidence-based surgical pathway that creates a multidisciplinary framework pre-, peri-, and postoperatively to provide patients with a quicker and safer recovery. Our findings demonstrate that higher use of the ERAS protocol in elective, inpatient TSAs is associated with decreased complications, in-hospital mortality, blood transfusion requirements, and LOS. The use of ERAS protocols in TSAs should be studied further, given its promising impact on perioperative outcomes and its ability to reduce mortality in patients. Future efforts should be made to create and integrate an official ERAS protocol for TSAs to optimize its effects.

## References

[1] Ljungqvist O, Young-Fadok T, Demartines N (2017). The history of Enhanced Recovery After Surgery and the ERAS Society. J Laparoendosc Adv Surg Tech A.

[2] Altman AD, Helpman L, McGee J, Samouëlian V, Auclair MH, Brar H, Nelson GS (2019). Enhanced Recovery After Surgery: implementing a new standard of surgical care. CMAJ.

[3] Grosh T, Elkassabany NM (2018). Enhanced recovery after shoulder arthroplasty. Anesthesiol Clin.

[4] McLaughlin DC, Cheah JW, Aleshi P, Zhang AL, Ma CB, Feeley BT (2018). Multimodal analgesia decreases opioid consumption after shoulder arthroplasty: a prospective cohort study. J Shoulder Elbow Surg.

[5] Memtsoudis SG, Fiasconaro M, Soffin EM (2020). Enhanced recovery after surgery components and perioperative outcomes: a nationwide observational study. Br J Anaesth.

[6] Ripollés-Melchor J, Ramírez-Rodríguez JM, Casans-Francés R (2019). Association between use of Enhanced Recovery After Surgery protocol and postoperative complications in colorectal surgery: the postoperative outcomes within Enhanced Recovery After Surgery Protocol (POWER) study. JAMA Surg.

[7] Wainwright TW, Immins T, Antonis JH, Hartley R, Middleton RG (2019). Enhanced Recovery After Surgery: concepts and application to total shoulder replacement. Orthop Nurs.

[8] Hu ZC, He LJ, Chen D (2019). An enhanced recovery after surgery program in orthopedic surgery: a systematic review and meta-analysis. J Orthop Surg Res.

[9] (2017). Development of an Enhanced Recovery After Surgery (ERAS) approach for lumbar spinal fusion. J Neurosurg Spine.

[10] Wainwright TW, Gill M, McDonald DA (2020). Consensus statement for perioperative care in total hip replacement and total knee replacement surgery: Enhanced Recovery After Surgery (ERAS(®)) Society recommendations. Acta Orthop.

[11] Wagner ER, Farley KX, Higgins I, Wilson JM, Daly CA, Gottschalk MB (2020). The incidence of shoulder arthroplasty: rise and future projections compared with hip and knee arthroplasty. J Shoulder Elbow Surg.

[12] Ma GC, Bradley KE, Jansson H, Feeley BT, Zhang AL, Ma CB (2021). Surgical complications after reverse total shoulder arthroplasty and total shoulder arthroplasty in the United States. J Am Acad Orthop Surg Glob Res Rev.

[13] Divo MJ, Martinez CH, Mannino DM (2014). Ageing and the epidemiology of multimorbidity. Eur Respir J.

[14] Padegimas EM, Maltenfort M, Lazarus MD, Ramsey ML, Williams GR, Namdari S (2015). Future patient demand for shoulder arthroplasty by younger patients: national projections. Clin Orthop Relat Res.

[15] Bixby EC, Boddapati V, Anderson MJ, Mueller JD, Jobin CM, Levine WN (2020). Trends in total shoulder arthroplasty from 2005 to 2018: lower complications rates and shorter lengths of stay despite patients with more comorbidities. JSES Int.

[16] Cox RM, Hendy BA, Gutman MJ, Sherman M, Abboud JA, Namdari S (2023). Utilization of comorbidity indices to predict discharge destination and complications following total shoulder arthroplasty. Shoulder Elbow.

[17] Lovy AJ, Keswani A, Beck C, Dowdell JE, Parsons BO (2017). Risk factors for and timing of adverse events after total shoulder arthroplasty. J Shoulder Elbow Surg.

[18] Farley KX, Wilson JM, Kumar A, Gottschalk MB, Daly C, Sanchez-Sotelo J, Wagner ER (2021). Prevalence of shoulder arthroplasty in the United States and the increasing burden of revision shoulder arthroplasty. JB JS Open Access.

[19] Paper PHDW (2025). Data that informs and performs. Premier Applied Sciences®, Premier Inc.

[20] Morgan ML, Davies-Jones GR, Ibrahim EF, Booker SJ, Bateman M, Tambe AA, Clark DI (2021). Introduction of an enhanced recovery programme for total shoulder arthroplasty: report of a novel pathway. BMJ Open Qual.

[21] Denard PJ, Lädermann A (2016). Immediate versus delayed passive range of motion following total shoulder arthroplasty. J Shoulder Elbow Surg.

[22] Sabesan VJ, Gilot G, Chatha K, Grunhut J, Brown S, Lavin AC (2022). The effect of an early mobilization rehabilitation protocol on outcomes after reverse shoulder arthroplasty. Semin Arthroplast.

[23] Tazreean R, Nelson G, Twomey R (2022). Early mobilization in enhanced recovery after surgery pathways: current evidence and recent advancements. J Comp Eff Res.

[24] Crain NA, Goharderakhshan RZ, Reddy NC, Apfel AM, Navarro RA (2021). The role of intraoperative urinary catheters on postoperative urinary retention after total joint arthroplasty: a multi-hospital retrospective study on 9,580 patients. Arch Bone Jt Surg.

[25] Geerlings SE (2016). Clinical presentations and epidemiology of urinary tract infections. Microbiol Spectr.

[26] Sipilä PN, Lindbohm JV, Batty GD (2023). Severe infection and risk of cardiovascular disease: a multicohort study. Circulation.

[27] Goon AK, Dines DM, Craig EV (2014). A clinical pathway for total shoulder arthroplasty-a pilot study. HSS J.

[28] Niesen AD, Hebl JR (2011). Multimodal clinical pathways, perineural catheters, and ultrasound-guided regional anesthesia: the anesthesiologist's repertoire for the 21st century. Minn Med.

[29] (2012). Practice guidelines for acute pain management in the perioperative setting: an updated report by the American Society of Anesthesiologists Task Force on Acute Pain Management. Anesthesiology.

[30] Kuo LT, Hsu WH, Chi CC, Yoo JC (2018). Tranexamic acid in total shoulder arthroplasty and reverse shoulder arthroplasty: a systematic review and meta-analysis. BMC Musculoskelet Disord.

